# Inhibition of O-GlcNAc transferase activates tumor-suppressor gene expression in tamoxifen-resistant breast cancer cells

**DOI:** 10.1038/s41598-020-74083-z

**Published:** 2020-10-12

**Authors:** Anna Barkovskaya, Kotryna Seip, Lina Prasmickaite, Ian G. Mills, Siver A. Moestue, Harri M. Itkonen

**Affiliations:** 1 Department of Circulation and Medical Imaging, NTNU, Trondheim, Norway; 2 Department of Tumor Biology, Institute for Cancer Research, Radiumhospital, Oslo University Hospital, Oslo, Norway; 3 Centre for Molecular Medicine Norway, Nordic European Molecular Biology Laboratory Partnership, Forskningsparken, University of Oslo, 0349 Oslo, Norway; 4 Nuffield Department of Surgical Sciences, University of Oxford, Oxford, UK; 5 Patrick G Johnston Centre for Cancer Research, Queen’s University of Belfast, Belfast, UK; 6 Department of Clinical and Molecular Medicine, NTNU, Trondheim, Norway; 7 Department of Health Sciences, Nord University, Bodø, Norway; 8 Department of Biochemistry and Developmental Biology, Faculty of Medicine, University of Helsinki, 00014 Helsinki, Finland; 9 Present Address: Department of Surgery, University of California San Francisco, California, USA

**Keywords:** Glycobiology, Breast cancer, Predictive markers, Biochemistry, Cancer, Biomarkers, Molecular biology, Chromatin, Epigenetics, Transcription

## Abstract

In this study, we probed the importance of O-GlcNAc transferase (OGT) activity for the survival of tamoxifen-sensitive (TamS) and tamoxifen-resistant (TamR) breast cancer cells. Tamoxifen is an antagonist of estrogen receptor (ERα), a transcription factor expressed in over 50% of breast cancers. ERα-positive breast cancers are successfully treated with tamoxifen; however, a significant number of patients develop tamoxifen-resistant disease. We show that in vitro development of tamoxifen-resistance is associated with increased sensitivity to the OGT small molecule inhibitor OSMI-1. Global transcriptome profiling revealed that TamS cells adapt to OSMI-1 treatment by increasing the expression of histone genes. This is known to mediate chromatin compaction. In contrast, TamR cells respond to OGT inhibition by activating the unfolded protein response and by significantly increasing *ERRFI1* expression. ERRFI1 is an endogenous inhibitor of ERBB-signaling, which is a known driver of tamoxifen-resistance. We show that *ERRFI1* is selectively downregulated in ERα-positive breast cancers and breast cancers driven by ERBB2. This likely occurs via promoter methylation. Finally, we show that increased *ERRFI1* expression is associated with extended survival in patients with ERα-positive tumors (p = 9.2e−8). In summary, we show that tamoxifen-resistance is associated with sensitivity to OSMI-1, and propose that this is explained in part through an epigenetic activation of the tumor-suppressor *ERRFI1* in response to OSMI-1 treatment.

## Introduction

Breast cancer is the most common cancer in women and over half of all breast cancers express estrogen receptor α (ERα), a nuclear hormone receptor^1^. Standard treatment in this patient group includes anti-ERα therapies, such as tamoxifen^2^. Resistance to this type of treatment is a significant challenge in the clinical setting, and results in the development of a more aggressive disease. One of the best understood mechanisms of tamoxifen-resistance is increased activity of receptor tyrosine kinases, most notably epidermal growth factor receptor (EGFR) and erb-b2 receptor tyrosine kinase 2 (ERBB2, Her2)^3–5^. Despite significant efforts, discovery of targeted therapies against tamoxifen-resistant breast cancer remains a significant challenge^6,7^.

O-GlcNAc transferase (OGT) has emerged as a candidate drug target in breast cancer. OGT is overexpressed in breast cancer patient tumor samples^8–11^, while genetic silencing of OGT decreases breast cancer cell proliferation both in vitro and in vivo^12,13^. In addition, it has been shown that OGT overexpression promotes tumor initiation in mouse models of breast cancer^14^. Conversely, OGT is not essential for the survival of post-mitotic normal mammalian cells^15–18^. These features position OGT as a candidate target for anti-breast cancer therapy.

OGT functions as a signaling hub that integrates nutrient status of the cell and modifies target proteins accordingly^19,20^. OGT’s co-substrate is produced via hexosamine biosynthetic pathway, which consumes glucose, glutamine, acetyl-CoA and ATP, the key metabolites involved in proliferation^21^. Depending on nutrient availability and other cues, OGT glycosylates intracellular proteins on serine and threonine residues, competing with protein kinases for substrates^22^. However, unlike hundreds of kinases, OGT is the solo enzyme catalyzing all protein glycosylation in the nucleus and cytoplasm. This unique role positions OGT as a major signaling hub.

Pharmacological inhibition of OGT represents a potential strategy to target this enzyme for cancer therapy. Several groups have developed compounds that alter O-GlcNAc signaling, but many of the compounds such as Alloxan (uracil mimic)^23^ and BAGDP (N-acetylgalactosamine mimic)^24^ require utilization of millimolar doses. An intriguing strategy is to use a synthetic carbohydrate precursor (Ac4-5SGlcNAc) that is converted into glycosyltransferase inhibitor within the cell^25^. This compound decreases aggressive phenotype of breast cancer cells^26,27^. However, a major limitation of this compound is that it additionally targets other carbohydrate processing enzymes. OSMI-1 is an OGT small molecule inhibitor that does not significantly affect other glycosyltransferases and is active in low micromolar doses^28^. We recently used OSMI-1 to demonstrate that triple-negative breast cancer cells are sensitive to OGT inhibition^29^. Overall, OSMI-1 represents an excellent tool compound to study OGT biology in vitro, and to discover possible strategies for anti-breast cancer therapy.

In this study, we used OSMI-1 to probe the importance of OGT activity for the survival of tamoxifen-resistant breast cancer cells in an isogenic cell line pair: Tamoxifen sensitive ERα-positive MCF7 cell line (TamS), and its tamoxifen-resistant derivative (TamR). We find that TamR cells show increased sensitivity to OSMI-1 relative to the parental line. Further, global transcriptome profiling shows that OSMI-1 treatment induces expression of the endogenous inhibitor of RTK-signaling, *ERRFI1*. The ERRFI1 promoter is hyper-methylated in breast cancer patient samples, which is associated with low expression levels and poor prognosis. Our findings suggest a link between OGT and ERRFI1 signaling, pointing to OGT as a possible therapeutic target in a significant subset of breast cancer patients.

## Results and discussion

### Tamoxifen resistant breast cancer cells are sensitive to OSMI-1

**Figure 1:**
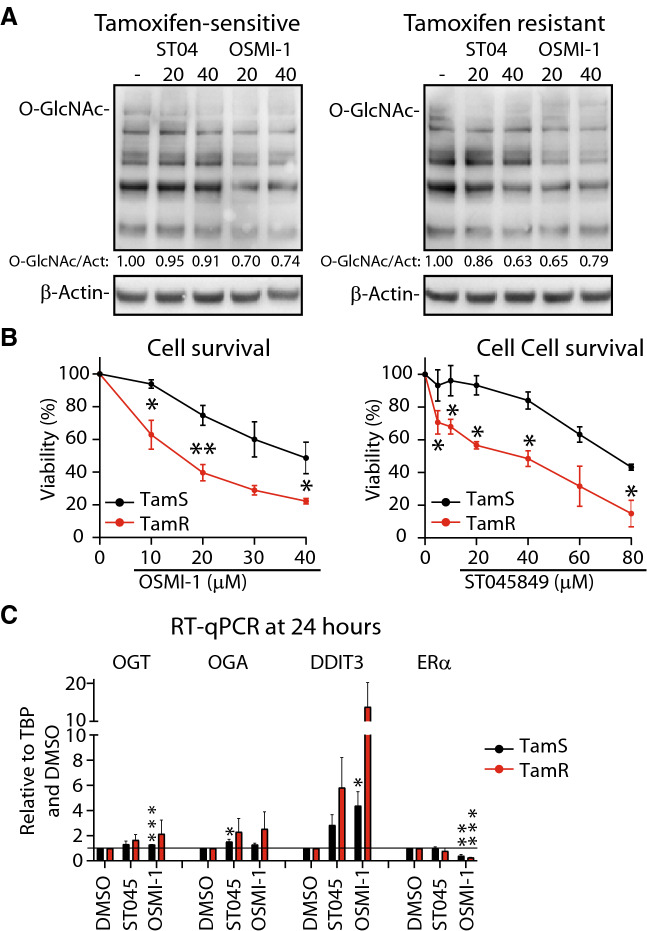
OGT inhibition reduces viability of breast cancer cells. **(A)** Total protein O-GlcNAcylation in TamS and TamR breast cancer cells after 24 h of treatment with OGT inhibitors—ST045849 or OSMI-1. Densitometry was used to determine the intensity of the signal. (**B)** Cell viability measured with MTS assay, following 72 h of treatment with increasing doses of either ST045849 or OSMI-1. An average of at least 3 biological replicates with SEM are shown. Significance of the data was evaluated using unpaired t-test, *-p ≤ 0.05, **-p ≤ 0.01. (**C)** RT-qPCR based evaluation of mRNA abundance following 24 h of OGT inhibition with either ST045849 or OSMI-1 at 40 μM dose. Average of three biological replicates with SEM are shown.

Here we compare the role of OGT in tamoxifen-sensitive MCF7 breast cancer cell line (TamS), and a previously established MCF7-derived tamoxifen-resistant cell line (TamR)^5^. First, we confirmed the tamoxifen-resistant phenotype of the TamR cells using relative cell number (DNA content) as a read-out (Suppl. Figure 1A). Notably, we did not observe significant differences in tamoxifen-sensitivity when using an MTS assay (relies on the activity of the NAD(P)H-dependent dehydrogenase enzymes, Suppl. Figure 1B). Next, we evaluated the overall O-GlcNAcylation and noted that both TamS and TamR have similar levels (Suppl. Figure 2A). The OGT small molecule inhibitor (OSMI-1) decreased total-O-GlcNAc by 30% in both cell lines (Fig. 1A). This effect was similar to treatment with another OGT inhibitor ST045849^30^. We also noted that OGT expression was increased in response to OSMI-1 treatment (Suppl. Figure 2B). Upregulation of OGT in response to OGT inhibition has been previously observed, and likely represents one of the compensation mechanisms that cells employ to restore O-GlcNAc homeostasis^31–33^. These data demonstrate on-target inhibition by both OSMI-1 and ST045849 in breast cancer cells.

**Figure 2:**
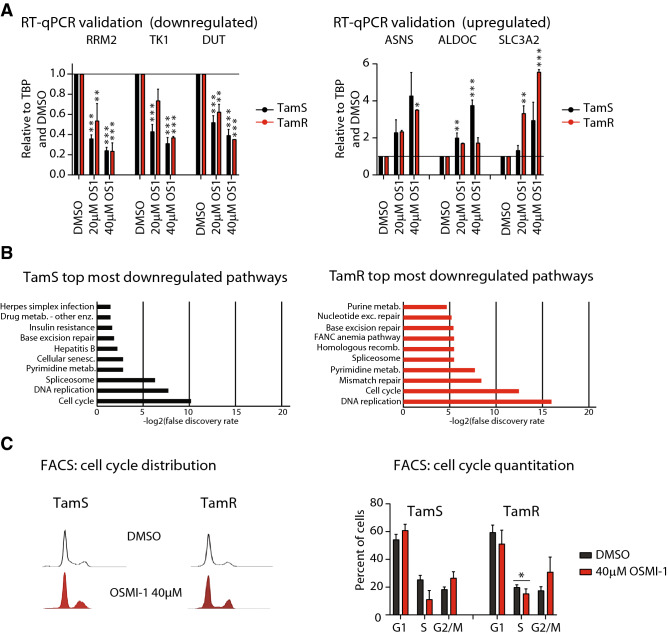
OGT inhibitor OSMI-1 decreases the percentage of breast cancer cells in S-Phase. **(A)** Validation of the microarray data using RT-qPCR. Cells were treated as indicated for 24 h, mRNA was isolated and used for RT-qPCR with select primers. Data shown is an average of four biological replicates with SEM and is normalized to DMSO treated control. (**B)** KEGG^55–57^ pathway enrichment analysis for the top most downregulated genes in TamS and TamR cells (Log2(FC) atleast − 0.5). (**C)** Cell cycle distribution of TamS and TamR cells after treatment with 40 μM OSMI-1 for 24 h. Data shown is an average of three biological replicates with SEM. Statistical analysis was performed using t-test, *-p ≤ 0.05.

Next, we assessed the importance of high OGT activity for proliferation of TamS and TamR cells using MTS assay relying on metabolic activity of cells and live-cell imaging to generate proliferation curves. OSMI-1 and ST045849 dose-dependently decreased viability of both cell lines as determined using the MTS assay (Fig. 1B). Interestingly, TamR cells were significantly more sensitive to both OSMI-1 and ST0145849 than the parental TamS cells, with OSMI-1-EC_50_ value of ~15 µM in TamR and ~ 40 µM in TamS. Live-cell imaging showed that OSMI-1 treatment blocks proliferation of TamS and TamR cells (Suppl. Figure 2C). Based on these data, we conclude that the metabolic activity of TamR cells is more sensitive to OGT inhibition, and moved on to explore the basis for this differential sensitivity.

OGT inhibition has been shown to significantly alter the transcriptional program of cells^31,34^, and we determined whether the differential sensitivity of TamR cells against OSMI-1 is reflected at the mRNA levels. OGT inhibition can suppress cancer cell proliferation in part through induction of DNA damaged-induced transcript 3 (DDIT3, also known as CHOP)^13^ and downregulation of mRNAs encoding for cell specific transcription factors that support cell proliferation^33^. One of the most prominent effects of OGT inhibition is compensatory upregulation of the gene encoding for OGT itself^31–33,35^. In accordance with these reports, we found that OSMI-1 causes increased expression of *OGT* and *DDIT3* in both TamS and TamR cells while ERα is downregulated (Fig. 1C). These effects are more prominent in TamR cells, suggesting that changes in the transcriptional program could underpin the differential sensitivity to OSMI-1.

### OSMI-1 induces transcriptional program indicative of chromatin silencing in TamS cells

To further explore the mechanism of increased sensitivity of TamR cells to OSMI-1 treatment, we performed a microarray transcriptomic analysis. We used RT-qPCR to validate the expression of genes that were among the top most downregulated (Ribonucleotide Reductase Regulatory Subunit M2 (*RRM2*), Thymidine Kinase 1 (*TK1*) and Deoxyuridine Triphosphatase (*DUT*)) and most upregulated (Asparagine Synthetase (*ASNS*), Aldolase, Fructose-Bisphosphate C (*ALDOC*) and Solute Carrier Family 3 Member 2 (*SLC3A2*), Suppl. Table 1). Expression of all six genes was differentially regulated by two to sixfold in response to OSMI-1 treatment, validating the microarray data (Fig. 2A).

Having established the quality of the microarray data, we performed pathway enrichment analysis to identify the key processes affected by the OSMI-1 treatment. OSMI-1 caused similar changes in the transcriptome of both cell lines and the top most upregulated pathways included steroid hormone biosynthesis and lysosomal degradation, which likely represent pro-survival adaptations to OSMI-1 treatment (Suppl. Table 1). Both TamS and TamR cells shared a number of significantly downregulated pathways including cell cycle, DNA replication and RNA processing (Suppl. Table 1, adjusted p-value < 0.05). In particular, we found the transcripts related to cell cycle progression to be highly enriched (Fig. 2B and Suppl. Table 1). To verify the impact of OGT inhibition on cell cycle regulation, we performed cell cycle analysis. OSMI-1 decreased the number of cells in S-Phase and also caused a modest, but not significant, accumulation of cells in the G2-M phase (Fig. 2C). These effects are in agreement with a previous study describing the importance of OGT for successful progression through mitosis^36^. However, cell cycle distribution in response to OSMI-1 was similar in both cell lines, and therefore cannot explain the increased sensitivity of TamR cells to the compound.

To explain the differential sensitivity of the cell lines to OGT inhibition, we focused on the mRNAs that were exclusively affected either in the TamS or TamR cells. We noted prominent downregulation of pathways related to DNA repair in TamS, and to a lesser extend also in TamR cells in response to OSMI-1 (Suppl. Table 1). The TamR-specific downregulated pathways were predominantly associated with metabolic processes such as amino acid degradation and fatty acid elongation (Suppl. Table 1). We have previously shown that inhibiting OGT leads to metabolic vulnerabilities by suppressing glycolytic activity, which sensitized cancer cells to inhibitors of oxidative phosphorylation^33^. This suggests that the enhanced sensitivity of TamR cells to OSMI-1 may be explained, in part, through the impaired ability to compensate for the metabolic stress induced by OGT inhibition. Tamoxifen treatment is known to impose a selection pressure for metabolic adaptations that support the emergence of resistance^37,38^, and in addition to targeting ERα, tamoxifen also suppresses oxygen consumption by blocking complex 1 of the electron transport chain, increases glycolysis and alters lipid metabolism^39^. It is possible that the increased sensitivity of TamR cells to OGT inhibitors is in part a side effect of these adaptations.

**Figure 3:**
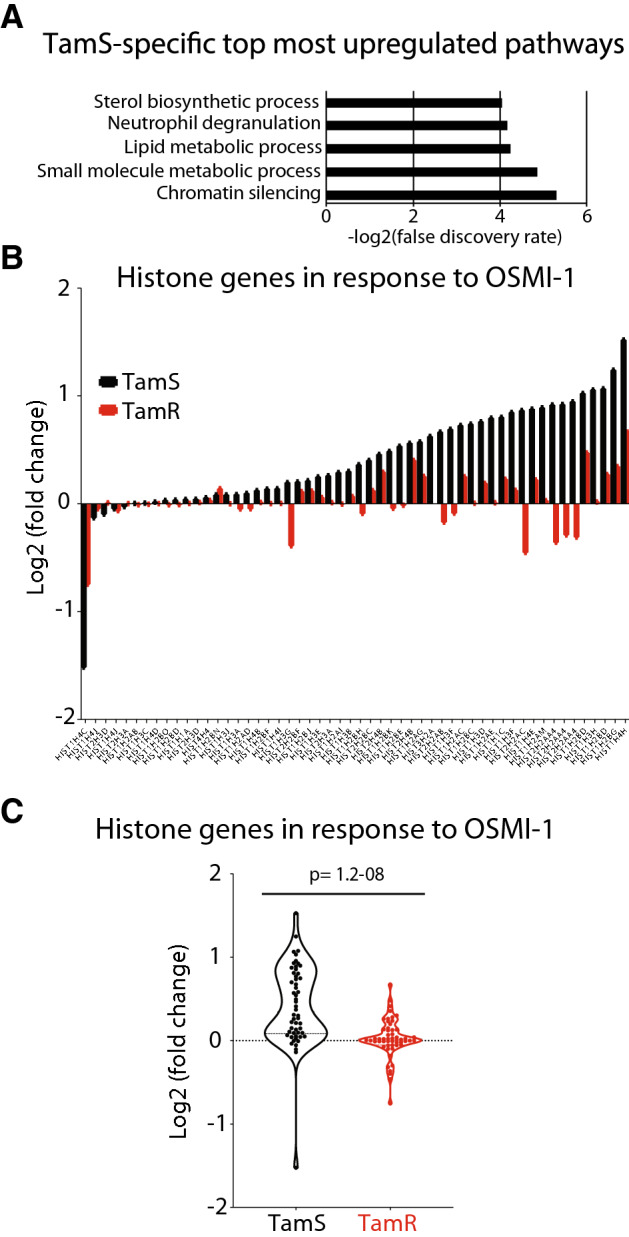
OGT inhibitor OSMI-1 induces transcriptional program indicative of chromatin silencing in tamoxifen-sensitive breast cancer cells. **(A)** KEGG^55–57^ pathway enrichment analysis for the mRNAs that were upregulated exclusively in TamS (Log2(FC) atleast ± 0.5). (**B)** Histone gene expression in TamS and TamR cells after OGT inhibition with 40 μM OSMI-1 for 24 h representing an average of three biological replicate experiments analyzed using microarrays. (**C)** Fold change in the expression of all detected histone genes after OGT inhibition in TamS and TamR cells (average of three biological replicate experiments analyzed using microarrays).

Next, we focused on the genes that were selectively upregulated in either of the cell lines as these could explain the differential sensitivity towards OSMI-1. We noted a strong activation of the transcriptional networks related to endoplasmic reticulum stress specifically in TamR cells (Suppl. Table 1). The most enriched pathway in TamS cells was ‘Chromatin silencing’, which was completely absent in TamR cells (Fig. 3A and Suppl. Table 1). When inspecting the mRNAs that explain the enrichment of this pathway, it became obvious that increased expression of the histone genes in response to OSMI-1 constitutes almost the entire gene list. Out of 54 histone mRNAs detected, 49 were increased in the TamS cells but remained largely unchanged in the TamR cells (Fig. 3B,C). This transcriptional response is the most striking differential response to OGT inhibition between TamS and TamR cells, and its mechanistic implications warrant further interrogation.

Increased expression of histone mRNAs has been previously demonstrated in response to bromodomain inhibitor-induced decrease in chromatin accessibility^40^. We hypothesized that if chromatin accessibility is altered in response to OGT inhibition in TamS cells, genes driven by super-enhancers would be most acutely affected by this treatment. To test this, we selected all super-enhancers in MCF7 cells ^41^ and assessed if the expression of associated mRNAs changes in response to OGT inhibition. Genes driven by super-enhancers were preferentially downregulated in response to OGT inhibition in the TamS cells when compared to the TamR cells (Suppl. Table 1). We have previously shown that chromatin O-GlcNAcylation overlaps with sites of active transcription and that OGT inhibition results in decreased chromatin accessibility in prostate cancer cells^42^. To fully elucidate the effects of OGT inhibition on chromatin accessibility in breast cancers cells, more direct assays such as FAIRE- or ATAC-seq are needed. While these experiments are essential to perform in follow-up studies, microarray data demonstrates that OGT inhibition results in a distinctive transcriptional response in TamS and TamR cells. By focusing on the differentially induced mRNAs, we may be able to identify those that have biologically meaningful functions in our model system and can additionally serve as prognostic breast cancer biomarkers.

### OSMI-1 induces expression of a negative regulator of receptor tyrosine kinase signaling in TamR cells

**Figure 4:**
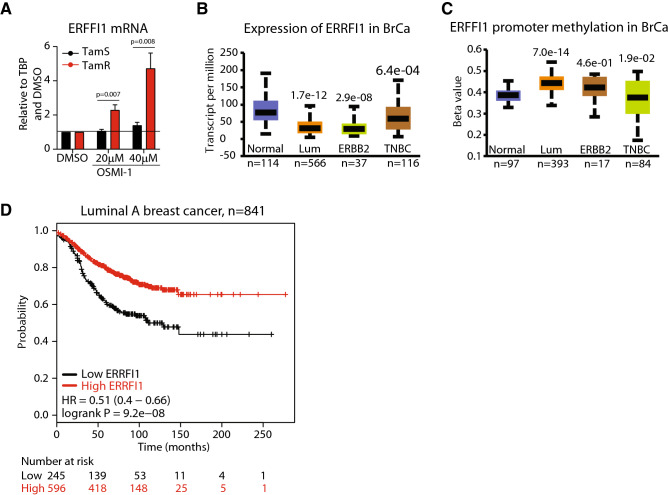
High expression of ERFFI is associated with positive prognosis in breast cancer patients. **(A)** RT-qPCR based evaluation of ERFFI1 expression after 24 h treatment with either DMSO or OSMI-1. Data shown is an average of four biological replicates with SEM and is normalized first to a house-keeping gene and then relative to DMSO treated control. Significance of the data was evaluated using unpaired t-test. (**B, C)** Expression and promoter methylation of ERFFI1 in breast cancer subclasses. The graphs were generated using UALCAN tool^58^ and the TCGA dataset. (**D)** Survival in breast cancer patients with low or high expression of ERFF1 mRNA. The plot was generated using Kaplan–Meier Plotter (mRNA gene chip, all probe sets for the gene, autoselect best cutoff and other settings as default)^59^.

Next, we identified the mRNAs that are selectively induced in TamR cells in response to OGT inhibition to identify potential tumor-suppressor genes that could function as disease biomarkers. Among five mRNAs that were most strongly induced in the TamR but not affected in TamS cells, we identified two genes, ERRFI1 (ERBB Receptor Feedback Inhibitor 1) and Ras Related Dexamethasone Induced 1 (RASD1) (Suppl. Table 1). OGT is important for the transcriptional regulation of certain genes^43^, and we therefore assessed if ERRFI1 and RASD1 genes carry an O-GlcNAc mark on their promoters using publicly available datasets^42,44^. Only ERRFI1 gene had a robust O-GlcNAc mark on its promoter (Suppl. Figure 3). Next, we used RT-qPCR to confirm that OSMI-1 treatment increases *ERRFI1* expression in TamR cells in a dose-dependent manner up to fivefold but does not have any effect in TamS cells (Fig. 4A). In addition, treatment with ST045849 and knockdown of OGT induced a significant increase in the ERRFI1 expression in TamR cells (Suppl. Figure 4). *ERRFI1* encodes a protein that functions as an endogenous inhibitor of ERBB-signaling—a well-established mechanism of tamoxifen-resistance^3–5^. In mouse models, deletion of ERRFI1 results in the development of spontaneous tumors^45,46^ while in human cells ERRFI1 decreases proliferation of cells that express high levels of epidermal growth factor receptor^47^. To gain further evidence of the tumor suppressor function of ERRFI1, we analyzed ERRFI1 gene dependency score using data available on the Cancer Dependency Map^48,49^. Depletion of ERFFI1 via CRISPR-mediated knockout increases the proliferation of almost all of the cancer cell lines included in this database (Suppl. Figure 5). Both the literature and CRISPR-data are consistent with ERRFI1 being a protein that negatively regulates proliferation of cells. However, additional experiments that go beyond the scope of this manuscript, characterization of TamS and TamR cells response to OGT inhibition, are required to assess if ERRFI1 is a *bona fide* tumor-suppressor. Nevertheless, these data suggest that the loss of ERFFI1 expression may be associated with the development of aggressive breast cancer.

We used publicly available datasets to evaluate *ERRFI1* expression and association with cancer progression. *ERRFI1* mRNA was downregulated in 17 separate breast cancer gene expression datasets available through Oncomine^50^, but was not prominently altered in other cancers, suggesting that this gene may be specifically associated with breast cancer (Suppl. Figure 6). Interestingly, *ERRFI1* expression was significantly lower in ERα-positive (luminal) breast cancer patient samples when compared to either normal tissue or triple-negative breast cancer (TNBC) samples (Fig. 4B). In addition, we noted that *ERRFI1* is downregulated in ERBB2-positive breast cancers, a disease driven by this receptor tyrosine kinase^51^. Overall, these data imply that *ERRFI1* functions as a tumor suppressor in breast cancers driven by ERα and/or ERRB-signaling.

We investigated the mechanistic basis for the loss of *ERRFI1* expression in breast cancer patient samples. *ERRFI1* expression can be controlled via DNA methylation^51^ and OGT is known to modify proteins that regulate chromatin accessibility^20^. *ERRFI1* promoter methylation was significantly higher in normal breast cancer tissue and TNBC patient samples when compared to the luminal breast cancer and ERBB2-positive breast cancer samples (Fig. 4C). High promoter methylation was associated with decreased *ERRFI1* expression (Fig. 4B,C). Next, we asked if the differential expression of *ERRFI1* has a prognostic value for breast cancer patients. We found that in luminal type A breast cancer patients, high expression of *ERRFI1* was very strongly associated with a positive outcome (9.2e−08, Fig. 4D). In addition, increased expression of *ERRFI1* was also significantly associated with good prognosis in patients treated with tamoxifen (Suppl. Figure 7).

To conclude, we report that tamoxifen-resistance is associated with increased sensitivity to OGT inhibition by OSMI-1 (Fig. 1B). We have previously demonstrated that TamS cells can be sensitized to OSMI-1 by tamoxifen^29^, and in this study we show that tamoxifen-resistant breast cancer cells are also dependent on high OGT activity. Even though we are not able to describe the specific mechanism, activation of the anti-proliferative transcriptional program specifically in endocrine-resistant breast cancer cells represents an intriguing strategy for cancer therapy. It has become apparent that OGT inhibitors can sensitize cancer cells to targeted therapies, such as inhibitors of phosphoinositide 3-kinase signaling^52^, glycolysis^53^ and proteasome^54^. It is not clear why a decrease in OGT activity sensitizes cancer cells to different treatments, and mechanistic understanding of this will enable more rational design of combination therapies with compounds targeting OGT. Results presented here suggest that targeting OGT may enhance the efficacy of ERBB receptor tyrosine kinase (RTK) inhibitors in cancer cells dependent on these RTKs. Based on our findings and previous data from others, we conclude that OGT functions as a context-dependent regulator of transcription.

## Materials and methods

### Cell culture

MCF7 cell line (referred to as TamS in this manuscript) was purchased from ATCC (Rockville, MD). TamS was cultured in DMEM (Sigma Aldrich) supplemented with 10% fetal bovine serum (FBS). TamR cells were a kind gift from Dr Julia Gee and Professor Robert I Nicholson (University of Cardiff), and have been previously described^5^. TamR cells were propagated in DMEM without phenol red (Thermo Fischer), supplemented with 5% charcoal stripped FBS. Cell lines were maintained in humidified incubators at 37 °C and were routinely tested for mycoplasma contamination.

### Western blotting

For western blotting analysis, TamS and TamR cells were seeded into 6-well plates, using 1.8 × 10^5^ cells per well in their respective media. Cells were treated with increasing concentrations of ST045849 (obtained from TimTec) and OSMI-1 (gift from Professor Suzanne Walker, Harvard Medical School, or obtained from Sigma Aldrich) the next day and collected on ice 24 h after the start of the treatment. Immunoblotting was performed using RL2 antibody (for total O-GlcNAcylation) (Abcam, ab2739), OGT (Cell Signalling, #5368) and β-actin antibody (Cell Signalling, #4967). Band intensity was quantified using ImageJ software.

### Cell cycle analysis

TamS and TamR cells were collected on ice following 24 h of treatment with 40 µM dose of OSMI-1 compound, or DMSO. Samples were immediately fixed in 100% ice-cold methanol and placed in − 20 C° for storage. Samples were washed in cold PBS and stained with 1.5 µg/ml Hoechst 33258 in PBS for 30 min at 37 °C. Cell cycle analysis was then performed on LSR II flow cytometer (BD Bioscience, San Jose, CA).

### MTS viability and crystal violet assays

Tamoxifen (Sigma, Prod. No. T5648) was freshly prepared just prior to the experiment. MTS (CellTiter 96 AQ_ueous_ Non-Radioactive Cell Proliferation Assay, Promega) was added to the cells at 1:5 dilution in growth media for 1–2 h at 37 °C. Absorbance at 490 nm was then measured using a Multilabel Counter Wallac plate reader. Results were corrected for background and normalized to DMSO-treated controls. For Crystal violet assay, cells were washed twice with PBS, fixed with ice cold 70% MeOH for 2 min followed by ice cold 100% MeOH for 10 min. After the cells dried out 0.05% crystal violet solution was applied for 10 min. After staining, cells were washed twice with distilled water and let to dry. For Crystal violet dissolution and measurement, 50 µL/well of 10% acetic acid was added, followed by 15 min de-stain on a plate shaker and absorbance was measured at 590 nM by the plate reader.

### Microarray data

Microarray profiling was performed at the NTNU Genomics Core Facility using Illumina HT-12 BeadChip arrays in biological triplicates.

### RNA isolation/RT-qPCR

Cells were collected following 24 h of treatment. mRNA was isolated using Illustra RNA spin mini kit (GE healthcare, Chicago, IL). cDNA was prepared using the qScript cDNA Synthesis Kit in accordance with the supplier’s instructions (Quanta Biosciences, Gaithersburg, MD). qPCR reactions were set up using 2.5 µl of 10 ng/µl stock cDNA, 2.5 µl of 1 µM forward and reverse primer mix and 5 µl of Fast SYBR Green master mix (Thermo Fischer Scientific). The following primers were used for gene expression analysis: OGT forward: CAGCATCCCAGCTCACTT, reverse: CAGCTTCACAGCTATGTCTTC; OGA forward: CGAGTGAACATTCCCATCACT, reverse: CCCAAAGGAGCACAGATGTT; DDIT3 forward: CTGGGGAATGACCACTCTGT, reverse: CTTGGCTGACTGAGGAGGAG; ERα forward: TGGGCTTACTGACCAACCTG, reverse: CCTGATCATGGAGGGTCAAA; RRM2 forward: TTTAGTGAGCTTAGCACAGCGGGA, reverse: AAATCTGCGTTGAAGCAGTGAGGC; TK1 forward: GCCGATGTTCTCAGGAAAAAGC, reverse: GCGAGTGTCTTTGGCATACTTG; DUT forward: CTATGGAGAAAGCTGTTGTGAAA, reverse: TTGCAGCCAAGCCTGACC; ASNS forward: ATCACTGTCGGGATGTACCC, reverse: CTTCAACAGAGTGGCAGCAA; ALDOC forward: CGTCCGAACCATCCAGGAT, reverse: CACCACACCCTTGTCAACCTT; SLC3A2 forward: ATTGGCCTGGATGCAGCTGC, reverse: ACAGCCCCTGGGATGTCAGG; ERRFI1 forward: TGCTGATGTGACCTCTGGAA, reverse: CCTTGTGTTGCTGGTTCCTA; TBP forward: GCCAGCTTCGGAGAGTTCTG, reverse: GCACGAAGTGCAATGGTCTTT. qPCR was run on a Bio-Rad CFX Connect Real Time PCR machine (Bio-Rad, Hercules, CA).

### Knockdown of OGT using siRNAs

Reverse siRNA knockdowns were performed using two OGT siRNAs: s16094 and s16095 (Thermo Fisher Scientific, Rockford, IL). 2.5 µL of 10 nM siRNA was mixed with 2 µl of Lipofectamine 3000 reagent (Invitrogen, Carlsbad, CA) in 360 µl Opti-MEM reduced serum medium (Thermo Fisher Scientific) per well. The mixture was incubated for 20 min in room temperature and placed into 6-well culture plates. The cells were added to the plates in 2 ml of respective cell culture media, using 1 × 10^5^ cells per well. Cells were collected for RNA extraction 72 h later.

## Supplementary information

Supplementary Information.

Supplementary Table 1.

## Data Availability

The data has been deposited to GEO with the accession number GSE148186.
