# Supplementary material for: Inhibition of O-GlcNAc transferase activates tumor-suppressor gene expression in tamoxifen-resistant breast cancer cells
Source: Sci Rep. 2020 Oct 12;10:16992. doi: 10.1038/s41598-020-74083-z (PMC7552408; doi:10.1038/s41598-020-74083-z)
Supplement: Supplementary file 1 — Supplementary Information. [file 41598_2020_74083_MOESM1_ESM.pdf]

Inhibition of O-GlcNAc transferase activates tumor-suppressor gene expression in tamoxifen-resistant breast cancer cells

Anna Barkovskaya, Kotryna Seip, Lina Prasmickaite, Ian G. Mills, Siver A. Moestue, Harri M. Itkonen

Supplementary Table legend and Supplementary Figures with figure legends

Supplementary table. TamS and TamR cells were treated with 40µM OSMI-1 and RNA was used for microarray profiling. Sheet 1) Differential gene expression based on microarray profiling of biological triplicate samples in TamS and TamR cell lines. **Sheet 2)** Biological Process pathway enrichment analysis using the STRING tool [1] for the top most up- and downregulated mRNAs based on sheet 1 (Log2(FC) atleast ±0.5). **Sheet 3)** Pathway enrichment analysis for the mRNAs downregulated in TamS and TamR cell lines (Log2(FC)<-0.25) using the Enrichr tool [2] and reporting KEGG pathways [3-5]. **Sheet 4)** Pathway enrichment analysis for the mRNAs downregulated exclusively in TamS and TamR cell lines (Log2(FC)<-0.25) using the Enrichr tool [2] and reporting KEGG pathways [3-5]. **Sheet 5)** Pathway enrichment analysis for the mRNAs upregulated exclusively in TamS and TamR cell lines (Log2(FC)>+0.5) using the STRING tool [1] and reporting Biological process. **Sheet 6)** Overlap between mRNAs downregulated logFC<-1.00 in TamS / TamR cells and mRNAs-driven by super enhancers in MCF7 cells as reported previously [6].

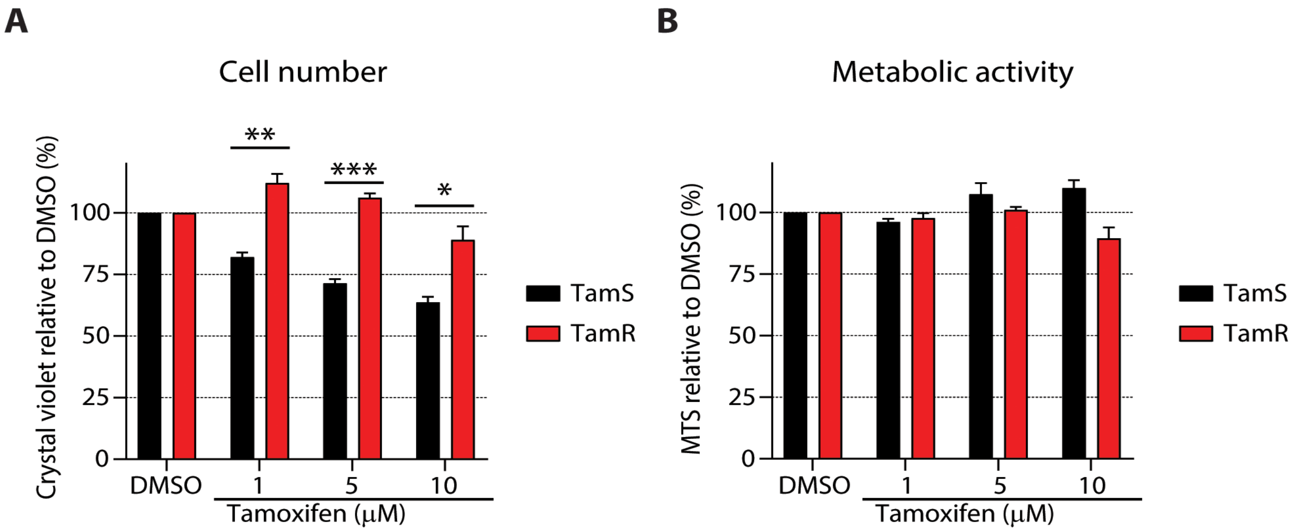

Supplementary Figure 1. Evaluation of tamoxifen-sensitivity of TamS and TamR cells. A) Cells were treated for three days and the relative number of cells was measured using crystal violet assay. Data shown is an average of 4 biological replicates with SEM. Student's t-test was used to evaluate statistical significance: *-p≤0.05, **-p≤0.01, ***-p≤0.001. **B)** Metabolic activity of cells was evaluated after three days of treatment. Data shown is an average of 3 biological replicates with SEM.

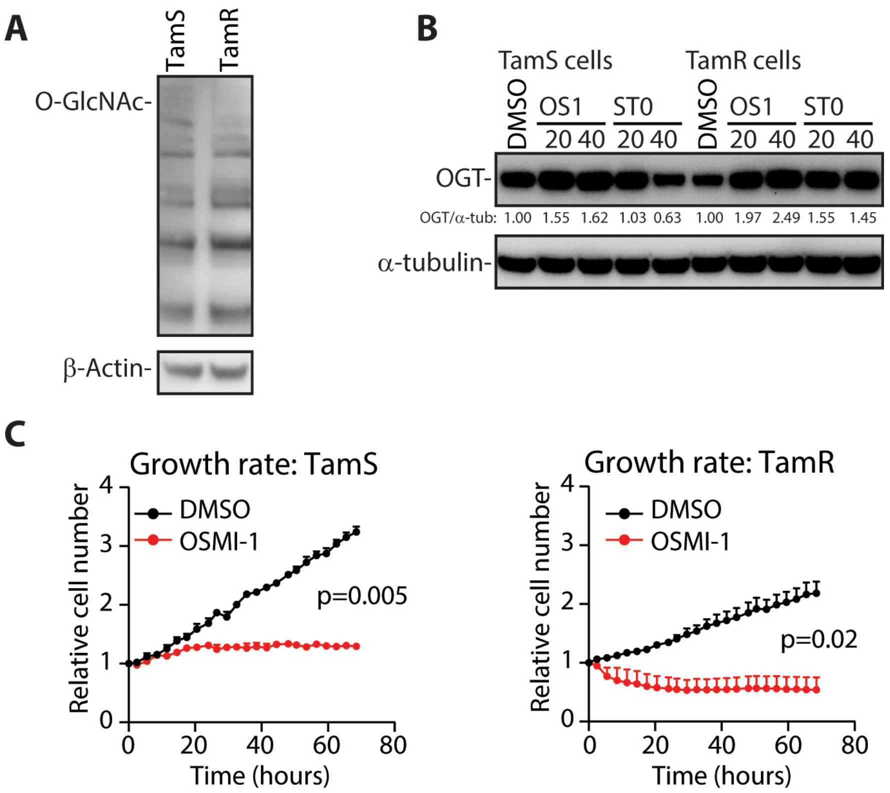

Supplementary Figure 2. OSMI-1 treatment leads to a complete growth-arrest in TamR cells. A) Comparison of the baseline O-GlcNAc levels in TamS and TamR cells based on western blot. **B)** Cells were treated as indicated for 24 hours and analyzed using western blot (OSMI-1= OS1, ST045849= ST0, doses µM). Data is representative of two biological replicates. **C)** Growth-rate of cells was followed using Incucyte live-cell imaging system. Relative cell number was normalized to density at the time of treatment. Data shown is an average of three biological replicates with SEM. T-test was used to assess statistical significance of the data at 72 hours.

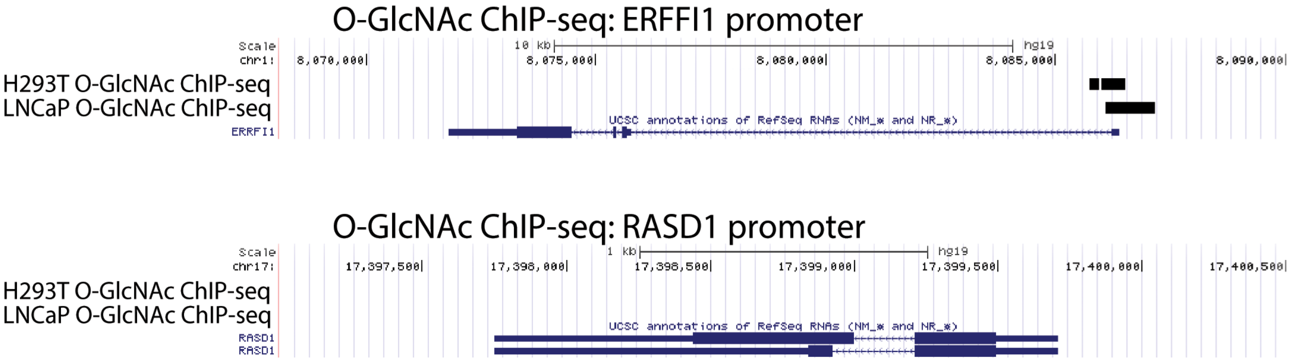

Supplementary Figure 3. ERRFI1 promoter shows enrichment for O-GlcNAc. University of California, Santa Cruz (UCSC) Genome Browser screenshot of the selected chromatin regions showing O-GlcNAc ChIP-seq data previously reported for LNCaP [7] and H293T cells [8].

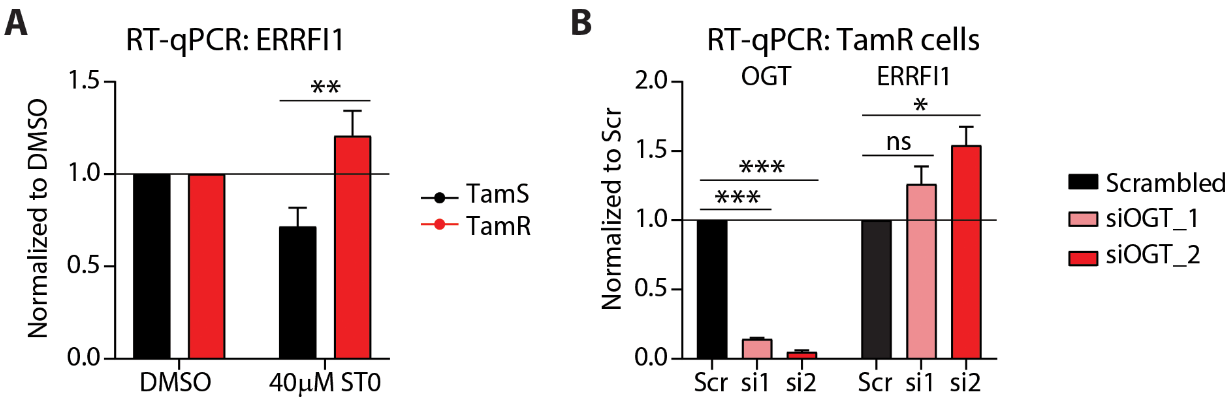

Supplementary Figure 4. OGT inhibition increases expression of ERRFI1 in TamR cells. Total RNA was isolated from the cell pellets using TRIzol® reagent (Life Technologies). 1µg of RNA was reverse transcribed using cDNA Synthesis Kit (Quanta Biosciences, Geithersburg, MD). Housekeeping gene (YARS) primers are as follows: forward: GCCTACCCAGATCCCTCAAAG and Reverse: ATGACCTCCTCTGGTTCTGAATTC; OGT and ERRFI1 primers, and RT-qPCR itself same as for the main figures. Data shown is an average of three biological replicates with SEM and student's t-test was used to evaluate statistical significance: *-$p \leq 0.05$, **-$p \leq 0.01$, ***-$p \leq 0.001$. **A)** Cells were treated as indicated for 24 hours and analyzed using RT-qPCR. **B)** Cells were transfected with either OGT-targeting siRNAs or scrambled control, and analyzed using RT-qPCR for mRNAs of interest.

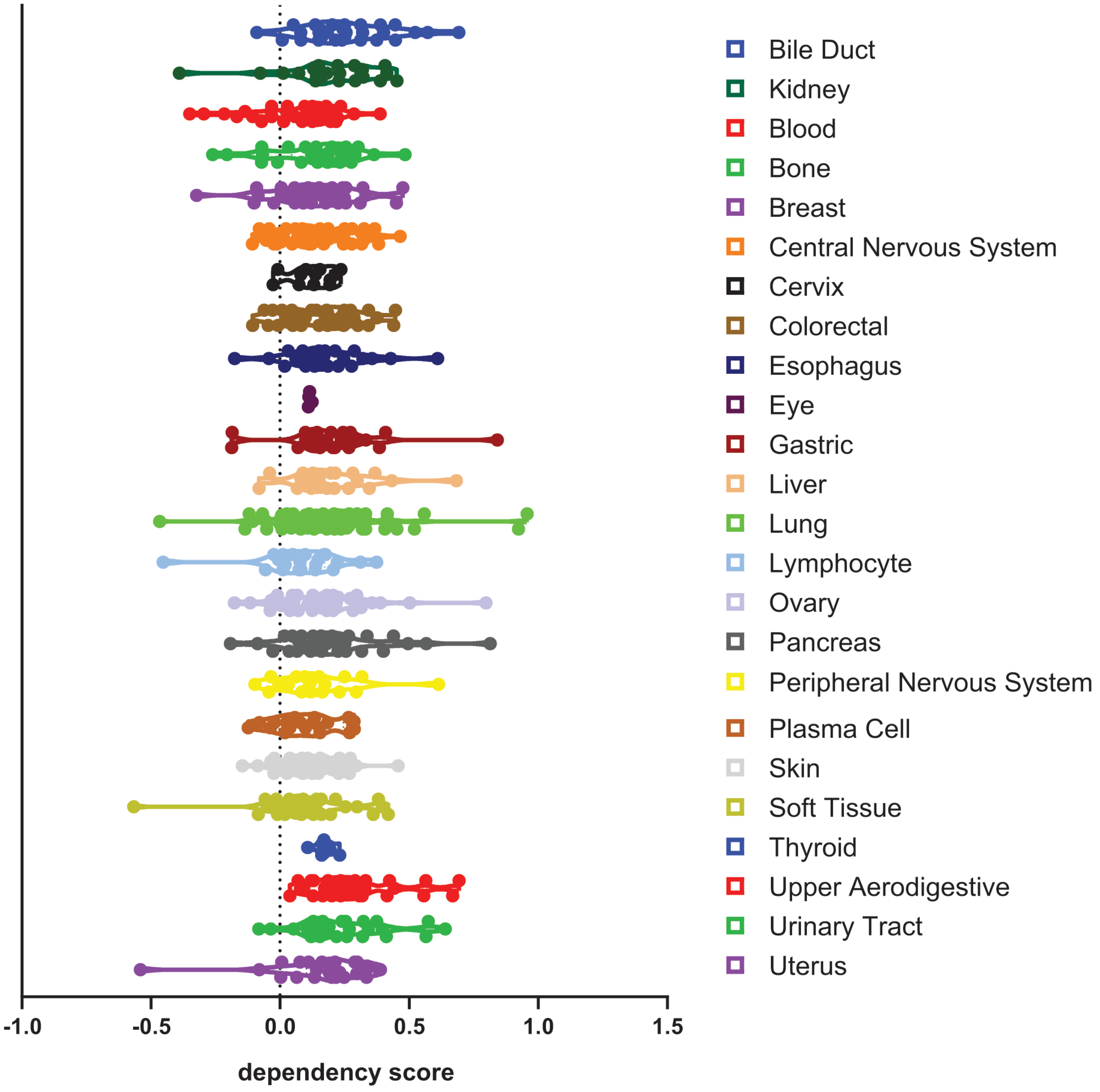

Supplementary Figure 5. CRISPR-mediated knockout of ERRFI1 promotes proliferation of cancer cell lines. ERRFI1 dependency score in cancer cell lines derived from different tissues. Data is acquired from depmap.org resource [9,10]. Data were collected from 342 cell lines where CRISPR knockout of ERRFI1 was induced. A score above 0 indicates that a knockout increases cell growth, while a score below 0 indicates growth inhibition. A score of -1 corresponds to the mean growth inhibition induced by the knockout of common essential genes. A score above 0 suggests tumor suppressive function of a gene.

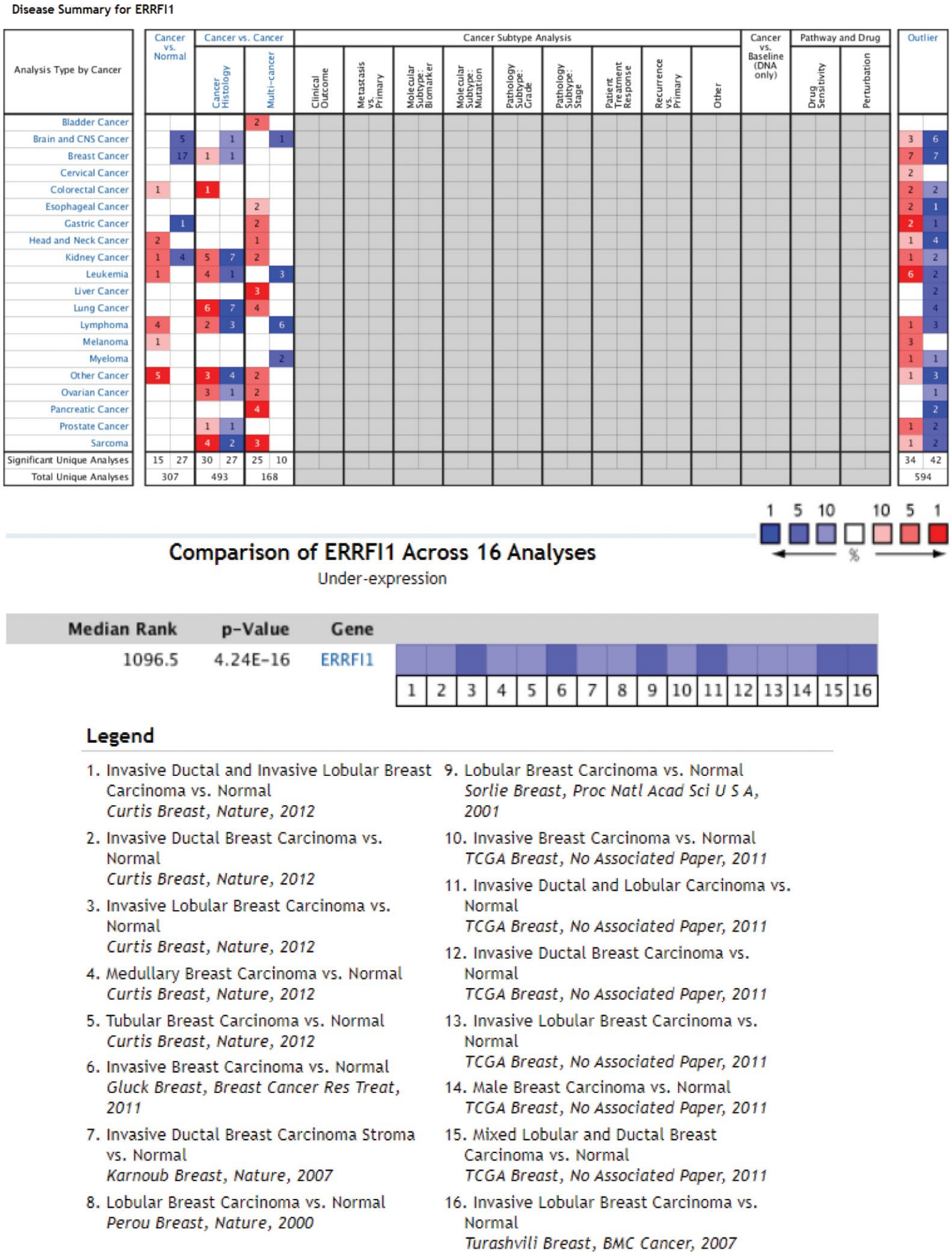

Supplementary Figure 6. ERRFI1 expression across different cancers. Oncomine database [11] was used to evaluate the expression of ERRFI1 in different cancers (Above). Expression of ERRFI1 in 16 different breast cancer microarray studies (below).

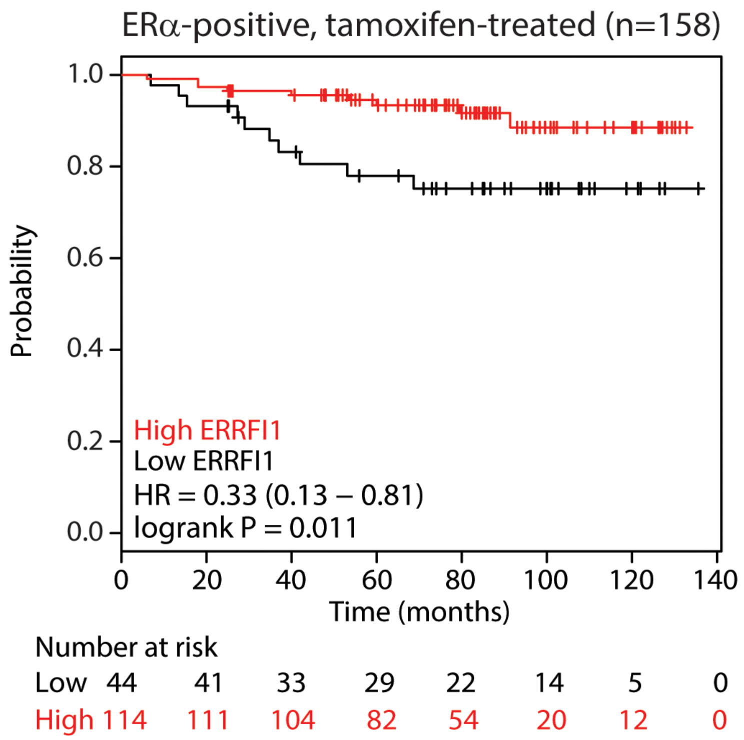

Supplementary Figure 7. High expression of ERFFI is associated with positive prognosis in breast cancer patients treated with tamoxifen. Survival of breast cancer patients with low or high expression of ERFF1 mRNA. The plot was generated using Kaplan-Meier Plotter (mRNA gene chip, all probe sets for the gene, autoselect best cutoff, ERα-positive cases based on gene expression, tamoxifen-only systemic treatment and other settings as default) [12].

A

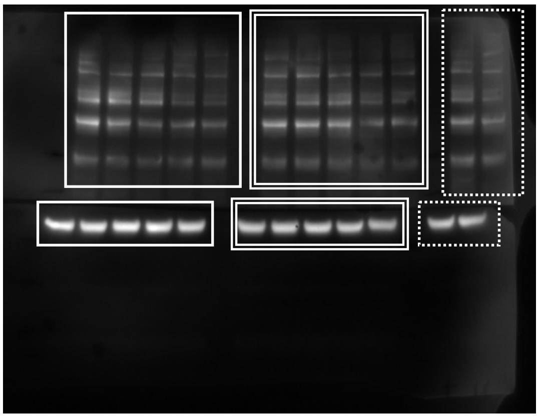

B

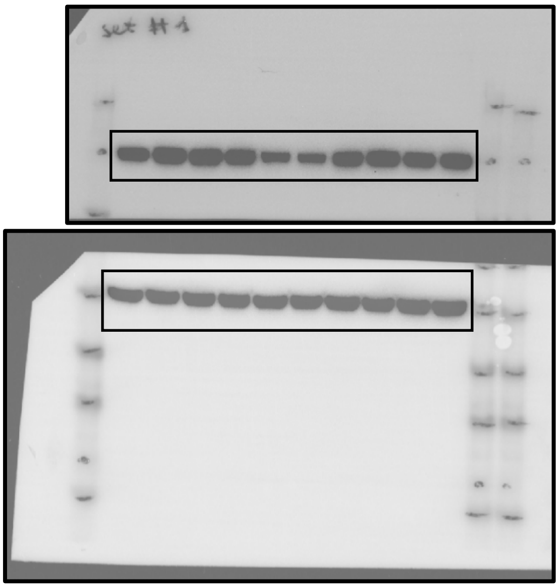

Supplementary Figure 8. Full size western blot images. Chemiluminescent images were generated using G-Box from Syngene™ (A) and Image lab software from BioRad™ (B). **A)** Original western blot data that was used to generate figure 1A (solid and double frames), and figure S2A (dotted frame). The western blot above is total-O-GlcNAc blot and below β-actin blot. **B)** Original western blot data that was used to generate figure S2B. The western blot above is an OGT blot and below β-tubulin blot.

References:

1 Szklarczyk, D. *et al.* STRING v11: protein-protein association networks with increased coverage, supporting functional discovery in genome-wide experimental datasets. *Nucleic Acids Res* **47**, D607-D613, doi:10.1093/nar/gky1131 (2019).

2 Kuleshov, M. V. *et al.* Enrichr: a comprehensive gene set enrichment analysis web server 2016 update. *Nucleic Acids Res* **44**, W90-97, doi:10.1093/nar/gkw377 (2016).

3 Kanehisa, M. & Goto, S. KEGG: kyoto encyclopedia of genes and genomes. *Nucleic acids research* **28**, 27-30, doi:10.1093/nar/28.1.27 (2000).

4 Kanehisa, M., Sato, Y., Furumichi, M., Morishima, K. & Tanabe, M. New approach for understanding genome variations in KEGG. *Nucleic acids research* **47**, D590-D595, doi:10.1093/nar/gky962 (2019).

5 Kanehisa, M. Toward understanding the origin and evolution of cellular organisms. *Protein science : a publication of the Protein Society* **28**, 1947-1951, doi:10.1002/pro.3715 (2019).

6 Khan, A. & Zhang, X. dbSUPER: a database of super-enhancers in mouse and human genome. *Nucleic Acids Res* **44**, D164-171, doi:10.1093/nar/gkv1002 (2016).

7 Itkonen, H. M. *et al.* High OGT activity is essential for MYC-driven proliferation of prostate cancer cells. *Theranostics* **9**, 2183-2197, doi:10.7150/thno.30834 (2019).

8 Deplus, R. *et al.* TET2 and TET3 regulate GlcNAcylation and H3K4 methylation through OGT and SET1/COMPASS. *EMBO J* **32**, 645-655, doi:10.1038/emboj.2012.357 (2013).

9 Meyers, R. M. *et al.* Computational correction of copy number effect improves specificity of CRISPR-Cas9 essentiality screens in cancer cells. *Nat Genet* **49**, 1779-1784, doi:10.1038/ng.3984 (2017).

10 Dempster, J. M., Rossen, J., Kazachkova, M., Pan, J., Kugener, G., Root, D. E., & Tsherniak, A. . Extracting Biological Insights from the Project Achilles Genome-Scale CRISPR Screens in Cancer Cell Lines. *BioRxiv*, doi:10.1101/720243 (2019).

11 Rhodes, D. R. *et al.* ONCOMINE: a cancer microarray database and integrated data-mining platform. *Neoplasia* **6**, 1-6, doi:10.1016/s1476-5586(04)80047-2 (2004).

12 Nagy, A., Lanczky, A., Menyhart, O. & Gyorffy, B. Validation of miRNA prognostic power in hepatocellular carcinoma using expression data of independent datasets. *Sci Rep* **8**, 9227, doi:10.1038/s41598-018-27521-y (2018).
